# Prince of Wales' Hospital, Cardiff

**Published:** 1918-03-09

**Authors:** 


### PRINCE OF WALES' HOSPITAL, CARDIFF.

### Opening by the Prince in Person.

The opening by the Prince of Wales of the Prince of Wales' Hospital for Limbless Sailors and Soldiers on February 20, 1918, was an unqualified success, although arranged very hurriedly. The hospital had been in use since May 1917, but there had been a special desire that the hospital should be formally opened by the Prince, for he consented to be patron when the idea was first mooted, and also granted special permission for the naming of the hospital after himself. The Prince's work in connection with the British Army in Italy had delayed the matter, but when he recently returned on leave he was again approached, and a week beforehand the Lord Mayor of Cardiff (who is vice-chairman of the Hospital Council and acting chairman during Mrs. Lloyd George's absence) received a message from Buckingham Palace that His Royal Highness would visit Cardiff on the following Wednesday to open the hospital.

£24,000 Raised in Three Days.

In January the Council decided to take steps, immediately the hospital had been formally opened, to raise an endowment fund of £100,000, so that the hospital might be used for civilian limbless cases when the demands arising from the war had lessened. The use for civilians was specially provided for by the trust deed. When the announcement of the forthcoming visit of the Prince was received, a deputation, consisting of the Lord Mayor of Cardiff and Colonel J. Lynn Thomas, C.B., C.M.G., was appointed to attend at Buckingham Palace on the following Saturday afternoon to inquire whether the Prince could see his way to receive gifts for the endowment fund whilst His Royal Highness was at the hospital on the occasion of the opening. Permission for this was graciously granted, and an appeal was accordingly drafted for insertion in the *Western Mail* and *South Wales Daily News* of Monday, February 18. The appeal was also telephoned to the *Manchester Guardian*, the *Liverpool Courier*, and *Liverpool Post* of the same date.

During Monday, promises for the endowment of three beds were received. An announcement to this effect was sent to the local papers for Tuesday, and it was very gratifying to receive, during Tuesday, eight other promises. On Wednesday morning other gifts were forthcoming, and at the opening ceremony the pleasing announcement was made that a fund of over £24,000 had been raised since the previous Monday morning; that is to say, in less than three days.

The Prince at the Hospital.

Several pleasing incidents occurred during the Prince's visit to the hospital, and the only thing to mar the proceedings in any way was the weather, which was poor, although the rain ceased for a short time during the opening ceremony. On arrival at the hospital the Prince was received, in the unavoidable absence of Mrs. Lloyd George (the chairman of the Council), by the Lord Mayor of Cardiff (the vice-chairman of the Council), and in the hall the following were presented to the Prince: The Commandant, Miss Clara Deacon, Colonel J. Lynn Thomas, C.B., C.M.G., and Colonel Sir Robert Jones, C.B., the senior consulting surgeons, Mr. John P. Cadogan and Mr. W. Percy Miles (chairman and vice-chairman of the Executive Committee), and the two donors of the hospital, Lord Aberdare, the treasurer, and Surgeon-General Sir James Hills-Johnes, V.C., G.C.B.

After signing the visitors' book, His Royal Highness proceeded to the recreation-room, where the other surgeons (Major Alwyn Smith, D.S.O., and Mr. James O. D. Wade, F.R.C.S.) and the secretary (Mr. Gilbert D. Shepherd) were presented by the Commandant, also Sister Love and Sister Blanning. After inspecting the nursing staff, the Prince inspected the patients, who were drawn up along the garden path, and then stood at one corner of the rock-garden whilst the whole of the patients who had been fitted with artificial legs walked up and down the various gradients to show how capable they were of climbing hills. The Prince appeared very much interested in the general lay-out of the garden, which is regarded as unique, and has proved most satisfactory.

The party then made a tour of the hospital, and proceeded to the flat roof over the recreation-room, where a platform had been erected at the corner overlooking the street, so that the vast crowd which filled West Grove and the streets adjoining might be able to see the ceremony. The Lord Mayor first called upon Mr. Cadogan, who, as chairman of the Executive Committee, asked the Prince formally to declare the hospital open. This he did in a few words, and the Lord Mayor called upon the secretary, Mr. Gilbert D. Shepherd, to announce the list of gifts. As each amount was mentioned the crowd cheered, and the Prince seemed pleased that the appeal should have received such a satisfactory response. Nearly all the donors were present, and personally presented their cheques to the Prince.

The crowd, led by the Wattstown male voice choir (consisting entirely of colliers), then sang one verse of the National Anthem and a verse of "God bless the Prince of Wales." Before leaving the flat roof, Mrs. W. Percy Miles (the chairman of the House Committee) presented the Prince with a beautifully bound copy of a book describing the history and work of the hospital.

The Prince then descended to the garden by the fire escape staircase, and inspected closely the workshops in which the limbs are made under the supervision of Mr. W. Blatchford. He also witnessed a demonstration of the capabilities of the Williams arm and the Blatchford working arm, with the Williams appliance fitted, and before leaving the hospital he inspected the officers' quarters and the men's billiard-room.

